# „Kompetenzen und ihre Entwicklung in der Arbeitswelt von Morgen: branchenunabhängig, individualisiert, verbunden, digitalisiert?“

**DOI:** 10.1007/s11612-021-00564-y

**Published:** 2021-02-17

**Authors:** Simone Kauffeld, Arnd Albrecht

**Affiliations:** Arbeits‑, Organisations- und Sozialpsychologie, Institut für Psychologie, TU Braunschweig, Spielmannstr. 19, 38106 Braunschweig, Deutschland

In einer sich wandelnden, dynamischen Arbeitswelt gewinnen neue Kompetenzprofile an großer Bedeutung. Sie ermöglichen, sich flexibel auf neue Anforderungen anzupassen und auch bei Unsicherheit adäquat zu agieren (Albrecht 2020; Kauffeld und Paulsen 2018). Unter Kompetenzen werden Fähigkeiten, Fertigkeiten und Wissensbeständen sowie Erfahrungen gesehen, die eine Person, ein Team oder eine Organisation bei der Bewältigung konkreter Arbeitsaufgaben handlungs- und reaktionsfähig machen und sich in der erfolgreichen Bewältigung von vor allem neuartigen Arbeitsaufgaben zeigen (Kauffeld 2006). Kompetenzen adressieren somit verschiedene Ebenen der physischen und psychischen Befähigung, die durch den Ansatz des Kompetenzmanagements verbunden werden. Kompetenzmanagement umfasst die systematische Analyse, Planung, Durchführung und Kontrolle einer Kompetenzentwicklung auf Grundlage eines Kompetenzmodells (Kauffeld und Paulsen 2018). In einem Kompetenzmodell werden Kompetenzen systematisiert, die strategisch und operationell relevant und somit entwickelt werden sollen. State of the Art des strategischen Personalmanagements ist die kompetenzbasierte Entwicklung von Mitarbeitenden. Sie stellt somit einen zentralen Baustein des HRM-Lebenszyklus dar. Die anhand der Kompetenzprofilen erbrachte Leistung erlaubt eine Einordnung in Gehalts- und Vergütungsstrukturen sowie der Entwicklungsprogramme. So werden schon in den Stellenbeschreibungen die hinterlegten Kompetenzen zur Suche von Fachkräften genutzt, die dann in den jeweiligen Karrierepfaden anhand von Kompetenzmusterentwicklung aufgebaut werden. Kompetenzen entwickeln sich auf Basis unterschiedlicher Lernprozesse, die sich hinsichtlich des Formalisierungsgrad anordnen lassen. Kompetenzentwicklung kann stark strukturiert (z. B. Trainings) sein, in den Arbeitsprozess eingebettet werden (z. B. Lerntandems) oder ungeplant und zufällig erfolgen (z. B. Lernen durch spontane Fehler) (Kauffeld und Paulsen 2018; Kauffeld und Frerichs 2018).

Lange dominierte im Weiterbildungsbereich die Annahme, dass die Lehrenden am besten wüssten, was und wie die Lernenden lernen sollten. Mit der kompetenzorientierten Wende in den 90er-Jahren (vgl. Kauffeld 2016) wurde auf die Bedeutung des arbeitsintegrierten Lernens jenseits formalisierter Curricula hingewiesen und hat viele Forschungs- und Praxisprojekte inspiriert (vgl. Ahrens und Molzberger; 2018; Bornewasser 2018; Hasebrook et al. 2018; Janneck und Hoppe 2018; Kauffeld und Frerichs 2018; Leimeister und David 2019; Bullinger-Hoffmann 2019; Knackstedt et al. 2020a, 2020b). Weiterbildung galt als chronisch verspätet (Staudt und Kriegesmann 1999). Der Transfer in die Arbeitswelt war und ist nur sicherzustellen, wenn förderliche und hinderliche Faktoren bei formalen (Kauffeld et al. 2008) und informellen Lernprozessen (z. B. Cerasoli et al. 2018) berücksichtigt werden. Das Zusammenspiel aus formellem und informellem Lernen kann dabei als agiles Lernkontinuum beschrieben werden. Nach einem formellen Lernprozess folgt ein vom Lerner selbst initiierter informeller Lernprozess (Lernen von und mit Anderen durch informellen Austausch, Selbstversuch oder Selbststudium Kortsch und Kauffeld 2019; Kortsch et al. 2019; Decius et al. 2019). Merkmale des Trainingsteilnehmenden wie beispielsweise die Motivation zum Transfer, die Transfervolition, aber auch Umgebungsfaktoren wie die Unterstützung von Kollegen und Vorgesetzten, die Möglichkeit von Belohnung oder Sanktion bei der Umsetzung des Gelernten wurden hierbei als entscheidende Faktoren für den Transfer, die über die Transfermotivation und die Transfervolition wirken, empirisch bestätigt (vgl. Grossman und Salas 2011; Seiberling und Kauffeld 2013; zusammenfassend Kauffeld 2016). Für das informelle Lernen wurde ein hoher Grad an Freiheit und Autonomie, sowie soziale Unterstützung als förderlich identifiziert (z. B. Cerasoli et al. 2018; Kortsch et al. 2019). Die Zufriedenheit nach einem formellen Training führt wiederum dazu, dass informelles Lernverhalten nach einem Training wahrscheinlich wird (Richter et al. 2020).

Bei sich verändernden Arbeitsbedingungen in der Agilen Welt entsteht das was zu lernen ist, zudem oft im laufenden Arbeitsprozess. Reflexionsprozesse sind dabei zentral (Faller et al. 2020). Arbeiten und Lernen finden so nicht mehr sequenziell, sondern gemeinsam und damit weitgehend gleichzeitig statt. Darüber hinaus sind Impulse von außen notwendig, um die Weiterentwicklung zu ermöglichen, wenn Wissen in der Organisation fehlt. Lernende können dann nach der Aufnahme entsprechender Impulse, Inhalte und Kontakte als Wissensträger nutzen, um die Organisation weiterzuentwickeln (Kauffeld 2016; Kauffeld und Paulsen 2018). Die digitale Transformation zielt auf die Einführung neuer Technologien und neuer Arten der Arbeitsorganisation (u. a. agiles Arbeiten) ab. Untrennbar verknüpft ist damit ein umfassender Lernprozess auf individueller und organisationaler Ebene, der gleichfalls durch steigende Digitalisierung und Agilität charakterisiert ist (Albrecht 2017a).

Einzelne Personen begeben sich auf Lernpfade und vernetzen unterschiedliche Lernformate, um Kompetenzen zu entwickeln (z. B. Poell et al. 2018; Kauffeld und Paulsen 2018). Der Lernende steht so nicht mehr am Ende des Lernprozesses, sondern auch am Anfang (Edelkraut und Mosig 2019; Kauffeld und Paulsen 2018). Dies geschieht nicht isoliert von einem sozialen und organisationalen Kontext. Das Arbeitsumfeld (Kauffeld et al. 2008; Kauffeld 2016), die Lernkultur und auch die Landeskultur (Richter et al. 2020) spielen eine entscheidende Rolle, ob und wie gelernt wird und inwieweit Gelerntes erhalten, verbessert und angewendet wird. Interessant ist die Frage, welche Kompetenzen bedeutsam werden und inwieweit künftig eine stark individualisierte Kompetenzentwicklung im Zuge eines organisationalen Kompetenzmanagements gelingen und unterstützt werden kann, vor dem Hintergrund einer stark dynamisierten Umwelt sowie in Verbindung mit künstlicher Intelligenz (Dellermann et al. 2019).

Im Zuge der stärkeren interdisziplinären Verflechtung und Zusammenarbeit werden immer mehr Kompetenzfelder verknüpft und verlangen zukünftig ein komplett neues Verständnis von Personalentwicklung hinsichtlich Stellenbeschreibungen und Karrierepfaden (Ko und Kirsch 2017). Zunehmend wird der Arbeitnehmer mehr in die Eigenverantwortung genommen, um seine Kompetenzen selbst auf zu bauen und weiter zu entwickeln im Sinne des lebenslangen Lernens. Der Vorteil des sogenannten *job crafting* ist dabei die hohe Eigenmotivation (Akkermann und Tims 2017). Zusätzlich hat sich der Anteil von Personen erhöht, die *remote working* schon vor der Corona-Pandemie durch das veränderte Arbeitsumfeld und den Bedeutungswandel von Organisationen genutzt haben (Albrecht 2017b; Felstead und Henseke 2017). Hier sind nicht nur technische Skills, sondern vielmehr selbst-organisatorische Kompetenzen stärker gefragt wie beispielsweise persönliche Initiative, um sich schneller und besser an Veränderungen anzupassen (Frese und Fay 2001; Kauffeld 2006). Beispielsweise muss sich das Führungsverständnis demnach viel stärker an den Technologisierungstrend einer immer stärker digitalisierten und globalisierten Welt hinsichtlich virtuellen Führens von Mitarbeitenden und Projektteammitgliedern angepasst werden (Albrecht 2016). Gleichzeitig muss aber dem Bedarf an emotionalen Kompetenzen im Rahmen der Begleitung und Führung von Mitarbeitenden in einer hoch komplexen und volatilen Umwelt Rechnung getragen werden, um den Arbeitnehmer wie in der COVID19-Arbeitsumgebung unlängst erkannt, vor Überlastungsschäden, zu schützen (Hillert et al. 2020).

In den Beiträgen wird der Frage nachgegangen, welche Kompetenzen zukünftig erforderlich sind oder wichtig werden. Gegenstand der Untersuchung ist die Kompetenzentwicklung in der modernen Arbeitswelt und wie sie gestaltet werden, wie das agile Konzept des Hybriden Projektmanagements im aktuellen Teil dieses Heft von **Albrecht** und **Albrecht** (2021, in diesem Heft) zeigt. Es werden konzeptionelle Ansätze ebenso beschrieben wie empirische Forschungsarbeiten und reflektierte Fallbeispiele zu Kompetenzanforderungen. Es wird aufgezeigt, wie „neue“ Kompetenzmanagementsysteme, die Möglichkeiten von Big Data und öffentlich zugänglichen Daten nutzen können, um den veränderten, schnellebigeren Arbeitswelten und damit den veränderten Stellenprofilen an die Mitarbeitenden nachzukommen (z. B. **Karwehl** und **Kauffeld** 2021, in diesem Heft). In verschiedenen Beiträgen werden neue Kompetenzanforderungen beschrieben oder tätigkeitsspezifisch Messinstrumente vorgestellt. Darüber hinaus finden sich in diesem Heft Ansätze zur Kompetenzentwicklung, die sich in der Praxis bewähren.

Welche neuen Ansätze zur Erfassung, Beschreibung und Systematisierung von Kompetenzanforderungen gibt es? **Karwehl** und **Kauffeld** (2021, in diesem Heft) geben in ihrem konzeptuellen Beitrag einen Überblick über etablierte und neue Wege im Kompetenzmanagement. Mit den Möglichkeiten von Big Data und prädiktiven Analyse wurde der Weg für ein neues Verständnis von Informationsverarbeitung geebnet, der sich für das Kompetenzmanagement nutzen lässt. Die oft langwierige Entwicklung von Kompetenzmodellen, die basierend auf der Organisationsstrategie regelmäßig angepasst werden müssten, ist, um flexibel auf veränderte Umfeldbedingungen in einem immer schnelleren globalen Wettbewerb zu reagieren, oft zu langsam. Einen datenbasierten Ansatz kann eine Erweiterung des bisherigen Vorgehens darstellen, um das Kompetenzmanagement zu unterstützen. In dem Beitrag werden neue technische Möglichkeiten, die sich aus dem HR Analytics ergeben, betrachten. Neben Chancen werden auch Risiken diskutiert.

**Lanwehr, Honsel** und **Wilms **(2021, in diesem Heft) betrachten öffentlich verfügbare Daten, um den Erfolg von Nachwuchsleistungszentren (NLZ) im deutschen Profifußball zu evaluieren. Damit bieten sie eine outputorientierte, ökonomisch und valide Alternative zu dem der aufwändigeren Expertenschätzungen, die für das Kompetenzmanagement in deutschen Profifußball bisher genutzt wird. Neben den Lösungen für das Kompetenzmanagement, die neue Daten und Datenzugänge mit sich bringen, werden neue Kompetenzanforderungen und Fähigkeitsprofile generiert, um den Bedarfen der Industrie und den Organisationen gerecht zu werden.

Die stärker dynamisierte Veränderung der Umwelt, Gesellschaft und der Industrie durch Megatrends kennzeichnen das 21. Jahrhundert im Sinne von Arbeit 4.0. Hier haben einzelne Autoren repräsentativ neuen Kompetenzanforderungen von Soft Skills und Hard Skills vorgestellt. So haben Rieth und Hagemann (2021, in diesem Heft) eine Arbeitsfeldbetrachtung zu veränderte Kompetenzanforderungen an Mitarbeitende infolge zunehmender Automatisierung durchgeführt. Der Untersuchungsgegenstand ist das interdisziplinäre Zusammenwirken von Fluglotsen und Piloten bei der Flugsicherung, in der in einem hochautomatisierten Umfeld. Hier wird das Eingreifen in eine M2M (Machine-to-Machine) -Welt Sinne der 4. Industriellen Revolution marginaler, aber dadurch entscheidender wird. Daraus wird abgeleitet, dass ein neuartiges Kompetenzmanagement mit dem permanenten Training neuer IT-Kompetenz und der Aufrechterhaltung und Anwendung bereits erlangter Fähigkeiten genauso wichtig ist wie die Adjustierung des Interface zwischen einem komplexen IT-System und den holistisch risikobeurteilenden kognitiven Fähigkeiten.

**Anke **und** Reingeisen **(2021, in diesem Heft) zeichnen das Kompetenzspektrum für Führungskräfte von agilen Softwareentwicklungsteams auf. Interessanterweise kamen dich meisten Entwicklungsimpulse aus der IT-Entwicklung. Der untersuchte Kompetenzrahmens von agilen Softwareentwicklungsteams. lassen folgende Schlüsse zu: Die Kompetenzanforderungen der Leitenden von Projekten sind durch vier tätigkeitsfeldspezifische Kompetenzen (Kenntnisse IT-Branche, Überblickswissen Softwareentwicklung, agile Arbeitsmethoden, Begeisterungsfähigkeit für Softwareentwicklung) and Performing und acht kontextunabhängige Dimensionen (Motivationsfähigkeit, Mitarbeiterförderung, Teamfähigkeit, Kommunikationsfähigkeit, Konfliktfähigkeit, Offenheit, Zeit- und Ressourcenmanagement, Proaktiver Umgang mit Fehlern).

Aufbauend auf der Forschung zu individuellem Grit („Biss“), d. h. der Kompetenz langfristige Ziele mit Passion und Beharrlichkeit zu verfolgen, beleuchten **Bernardy** und **Antoni** (2021, in diesem Heft) die Rolle von Team Grit, im Rahmen von dynamischen Innovationsprozessen. V. a. werden dabei Ansteckungs- und Crossover-Prozesse als Mechanismen betrachtet, die Team Grit beeinflussen. Empfehlungen zum Aufbau von Teamkompetenzen zur Stärkung des Team Grits und zur innovativen Leistungsteigerung im Team, werden abgeleitet.

Auf die Kompetenzen von Trainer/innen in v. a. formalen Lernsettings gehen **Grohmann, Schulte** und **Kauffeld** (2021, in diesem Heft) in ihrem Beitrag ein. Sie stellen einen mehrdimensionaler und zeitlich effizienter Kurzfragebogen zur Beurteilung von Trainer/innenkompetenzen in beruflichen Weiterbildungsmaßnahmen vor und überprüfen in drei Studien. Theoretische und praktische Implikationen für den Einsatz in Unternehmen werden aufgezeigt.

**Muschala **und** Katzner **(2021, in diesem Heft) gehen in ihrem Beitrag der Frage der psychischen (Arbeits)Fähigkeiten bei akademisch qualifizierten Berufseinsteigern mit und ohne (psychischen) Gesundheitsproblemen nach. Gehen körperliche oder psychische Erkrankungen mit Defiziten in (welchen) Fähigkeitsdimensionen einher? Dabei zeigte sich, dass Studierende mit psychischen Gesundheitsproblemen stärkere Prüfungsängste und geringere Selbstwirksamkeit im Vergleich zu Studierenden mit körperlichen Gesundheitsproblemen oder Gesunden sowie Einschränkungen in sozialen Fähigkeiten hatten, was sich jedoch kaum in Kompetenz- und inhaltsbezogenen Fähigkeiten widerspiegelt.

Studierende mit Gesundheitsproblemen hatten keine generell schwächeren Fähigkeiten. Studierende mit psychischen Gesundheitsproblemen.

Neben der Kompetenzdiagnose beschäftigen sich einige Beiträge mit der Kompetenzentwicklung – besonders in agilen Lernprozessen. **Jungclaus **und** Schaper **(2021, in diesem Heft) stellen eine theoriegeleitete Analyse der Wirkprinzipien des Agilen Sprintlernens, das als ein neuartiger Gestaltungsansatz für die arbeitsbezogene Kompetenzentwicklung gelten kann, vor. Die Autoren identifizieren neun zentrale Gestaltungs- und Wirkungselemente Zielorientierung und Zielverfolgung, Transparenz und Klarheit, Planung des Lernens, Eigenaktivität und Eigenverantwortung, Feedback, Selbstreflexion, Autonomie, Kompetenzerleben und soziale Eingebundenheit, die für den Erfolg des Agilen Sprintlernens verantwortlich gemacht werden können.

**Weihrauch, Wolff, Söger, Nitzsch & Konari** (2021, in diesem Heft) liefern in Ihrem Beitrag Hinweise, wie durch ein Trainingsformat ein erfolgreiches Netzwerk aufgebaut, gepflegt und genutzt werden kann. Interpersonelle und soziale Kompetenz sind dabei für den Aufbau eines strategischen Beziehungsnetzwerks und die Einbindung von den vielschichtigen Stakeholdergruppe unerlässlich. Die Konzeption, Durchführung und Evaluation eines Trainings hinsichtlich zentraler Maße: netzwerkbasiertes Wissen, Selbstreflexion und Verhalten, sowie eine Zunahme an Kontakten in beruflichen Social Media, steht im Mittelpunkt des Beitrages.

**Genkova und Kruse **(2021, in diesem Heft) untersuchen in ihrer Arbeit die Erfahrung aus Entsendungen von Mitarbeitende ins Ausland. So kann gezeigt werden, dass kulturelle Kompetenz in Abhängigkeit der Entsendungsdauer und dem Austausch mit Kulturvertretern steht, und intrapersonelle Skills wie Widerstandsfähigkeit erhöht werden. Daraus leiten die Autoren ab, dass zum Aufbau von Kompetenzen in (internationalen) Führungspositionen und zur persönlichen Weiterentwicklung Expatriierung geeignet sind, allerdings nicht für alle Positionen und Karrierepfade von Mitarbeitenden eines Unternehmens.

In einem weiteren Beitrag werden intrapersonellen Kompetenzen wie Resilienz betrachtet. **Ladinig** (2021, in diesem Heft) hat Untersuchungen zur Achtsamkeit am Beispiel von Qi-Gong-basierten Interventionen am Arbeitsplatz durchgeführt. Die Autorin leitet von den Ergebnissen neben der Steigerung von Soft-Skills eine Verbesserung des allgemeinen körperlichen Gesundheitszustands ab.

Im offenen Teil des Heftes stellen **Mander, Hellert** und **Antoni **(2021, in diesem Heft) in ihrem Beitrag Selbstführungsstrategien zur Bewältigung von Flexibilitätsanforderungen digitaler Arbeit mit hohem Zeit- Orts- und Handlungsspielraum vor. Diese qualitative Studie zum Self-Leadership zeigt die Implikation mit dem Umgang mit (neuen) Kommunikationsformen und der hybriden Beruf-Privatwelt, die an Aktualität durch COVID 19 gewonnen hat.

**Müller-Frommeyer und Kauffeld** (2021, in diesem Heft) betrachten wiederkehrende Formen der sozialen Interaktion in Organisationen, die für den persönlichen und organisationalen Erfolg bedeutsam sind. Das Potenzial der Analyse von impliziter (d. h., die individuelle und simultane Äußerung von unbewusst genutzten Funktionsworten wie Pronomen, Artikel und Präpositionen) und expliziter (d. h., die übergeordnete(n) Funktion(en) einer Aussage) Kommunikation in organisationalen Interaktionen wird dargestellt.

Mit dem Thementeil dieses Heftes werden verschiedene Fragestellungen zum Kompetenzmanagement, zur Kompetenzmessung und zur Kompetenzentwicklung beantwortet. Gelichzeitig bleiben viele Fragestellungen unbeantwortet: Welche Kompetenzen gewinnen branchenübergreifend an Bedeutung? Welche Kompetenzen sind historisch geschlechtsattributiert und bedürfen hinsichtlich der Leistungsüberprüfung einer Überarbeitung? Inwieweit wird sich bei der Entwicklung von Mitarbeitenden auf ein kompetenzbasiertes Management verlassen und wie können agile Ansätze das Kompetenzmanagement verändern? Wie kann ein Kompetenzmanagementsystem individuelle Kompetenzentwicklung stärker berücksichtigen? Welche Bedeutung können Ansätze der Big Data und des Maschinellen Lernens auf die Diagnose von Kompetenzen, das Matching auf Lernpfade und zu Kompetenzentwicklungsmaßnahmen einnehmen. Erste vielversprechende Ansätze sind hier in der Entwicklung. Welche Rolle spielen generell digitale Tools bei der Kompetenzentwicklung? Welche neuen Ansätze des Kompetenzmanagement gibt es, die flexibler auf veränderte Kompetenzanforderung einhergehen oder Verknüpfungen zu anderen Ansätzen aufweisen (z. B. Gesundheitsmanagement, Onboarding)?

Globale Megatrends wie Digitalisierung, Internationalisierung, Individualisierung und demografischer Wandel als auch der Einfluss der Corona-Pandemie hat zu einer größeren Akzeptanz aber auch Diskussion von digitalen und intrapersonellen Kompetenzen einerseits geführt, zum anderen geraten digitale Kompetenzentwicklungsmöglichkeiten in den Fokus. Es bleibt zu hoffen, dass die Bereitschaft zunimmt, das zukünftige Portfolio an Kompetenzen und Kompetenzentwicklungsmaßnahmen für eine zukunftssichere Employability weiter zu untersuchen. Sowohl Grundlagenforschung aus der Psychologie und Soziologie als auch Erkenntnisse aus der angewandten Umsetzung von Ansätzen zum Kompetenzmanagement in Unternehmen und Organisationseinheiten können dazu beitragen.
